# Giant Parathyroid Adenoma-Associated Fracture, Not All Lytic Bone Lesions are *Cancer*: A Case-Based Review

**DOI:** 10.1155/2022/3969542

**Published:** 2022-01-29

**Authors:** Jose C. Alvarez-Payares, Marcel E. Ribero, Sara Ramírez-Urrea, María C. Fragozo-Ramos, Jose E. Agámez-Gómez, Alejandro Román-González, Luis F. Arias, Roberto Benavides Arenas, Fernando López-Urbano

**Affiliations:** ^1^Internal Medicine Department, Universidad de Antioquia, Hospital Universitario, San Vicente Fundación, Medellín, Colombia; ^2^General Medicine, Fundación Universitaria San Martín, Medellín, Colombia; ^3^Endocrinology and Metabolism Section, School of Medicine, Universidad de Antioquia, Endocrinology Department, Hospital San Vicente Fundación, Medellín, Colombia; ^4^General Medicine, Universidad de Antioquia, IPS UniversitariaClínicaLeón XIII, Medellín, Colombia; ^5^Internal Medicine Department, Endocrinology and Metabolism Section, School of Medicine, Universidad de Antioquia, Endocrinology Department, Hospital San Vicente Fundación, Medellín, Colombia; ^6^Pathology Departament, Universidad de Antioquia, Hospital Universitario San Vicente Fundación, Medellín, Colombia

## Abstract

**Introduction:**

Due to the early diagnosis of primary hyperparathyroidism the musculoskeletal manifestations of this disease are becoming less frequent. When this disease manifests secondary to a giant adenoma, it presents with more aggressive symptoms and can have important repercussions such as the hungry bone syndrome after parathyroidectomy. There are few reported cases of hyperparathyroidism secondary to a giant adenoma in the literature, as the presence of a brown tumor is often misinterpreted as a metastatic lesion from an unknown primary tumor.

**Methods:**

We describe a case and performed a literature review to identify all case reports. A literature search was carried out on PubMed/MEDLINE and EMBASE bibliographic databases. All available studies from May 2009 to May 2021 were included. Data were tabulated, and outcomes were cumulatively analyzed.

**Results:**

Twenty-four cases of primary hyperparathyroidism due to giant adenoma have been described; the majority were women, with a mean age of 52 years. They presented with heterogeneous symptoms such as palpable nodules (45%), bone pain (33%), brown tumor (12.5%), asymptomatic (12.5%), metabolic profile with a mean calcemia of 13.8 mg/dL, PTH 1109 ng/L, and mean tumor weight of 47.24 g.

**Conclusion:**

Primary hyperparathyroidism due to giant adenoma increases the risk of developing potentially serious postoperative complications such as hungry bone syndrome. This implies the need of implementing preventive measures comprising administration of intravenous zoledronic acid and early supplementation of oral calcium to prevent complications after resection.

## 1. Introduction

Primary hyperparathyroidism (PHPT) arises from the excessive function of parathyroid glands, secondary to an adenoma, gland hyperplasia, or parathyroid carcinoma [1]. This clinical entity is the third endocrine disease in frequency, after diabetes and thyroid disease, and is two to three times more frequent in women than men [2].

Parathyroid adenomas (PTA) have been described to be the cause for up to 85% of PHPT [3]. A PTA with a weight of >3.5 g are classified as giant parathyroid adenomas (GPTA) [3, 4]. However, it is important to note that the measurement may only be obtained after surgical excision, which is an additional diagnostic challenge.

Nowadays, PHPT is less associated with severe skeletal features due to prompt diagnosis. Nonetheless, most studies published in developing countries previously report these kinds of lesions in half of the patients (52.9%) [5]. Bone disease is considered an important risk factor for immediate postoperative, symptomatic, and prolonged hypocalcemia requiring a readmission for intravenous calcium. This phenomenon is called hungry bone syndrome (HBS) [6] and occurs because of a rapid drop in serum parathyroid hormone (PTH) level after a long period of sustained elevation.

We present the case of a patient with GPTA associated with classical hyperparathyroidism features, diagnostic tests, complications, and treatment. Furthermore, we performed a case-based literature review about PHPT cases secondary to GPTA.

## 2. Methods

**Figure 1:**
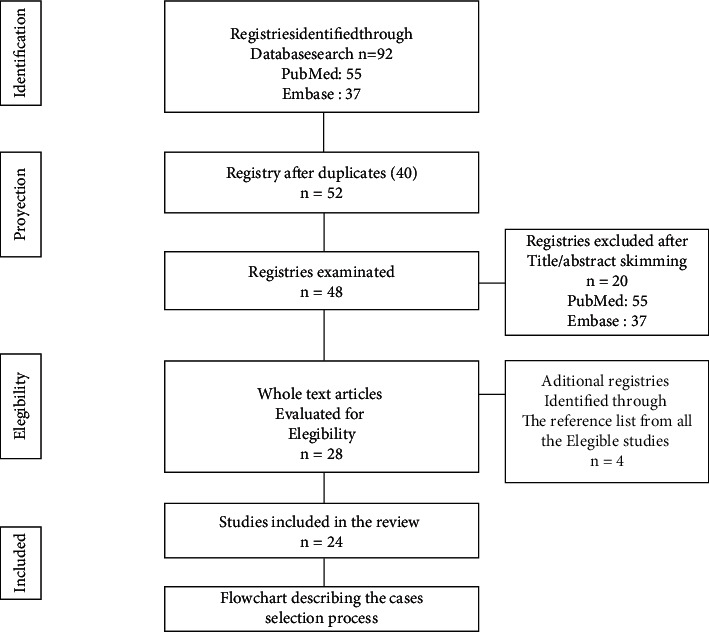
Flowchart describing the case selection process.

A literature review was performed in PubMed and EMBASE databases using the keywords, “primary hyperparathyroidism”, “giant parathyroid adenoma”, “hungry bone syndrome”, “pseudogout”, “brown tumor”. Articles published in English, Portuguese, or Spanish from May 2009 to May 2021 were included as the first reported case regarding the giant parathyroid adenomas was published in 2009. A total of 92 articles matched the search criteria of which, full texts were included, regardless of the methods used (Figure 1). The clinical, demographical, laboratory, post-operatory complications, and treatment, were analyzed.

## 3. Case Presentation

A 55-year-old female patient was referred from the outpatient clinic due to a four-year history of mechanical pain in the right shoulder, which progressed to the knees, clavicle region, hips, and lumbar spine. The patient also mentioned an exacerbation in pain and loss of strength in the right upper limb during the month before consultation. The patient had also undergone a cholecystectomy six months before admission as well as had a past medical history of arterial hypertension and osteoarthritis in medical management.

**Figure 2:**
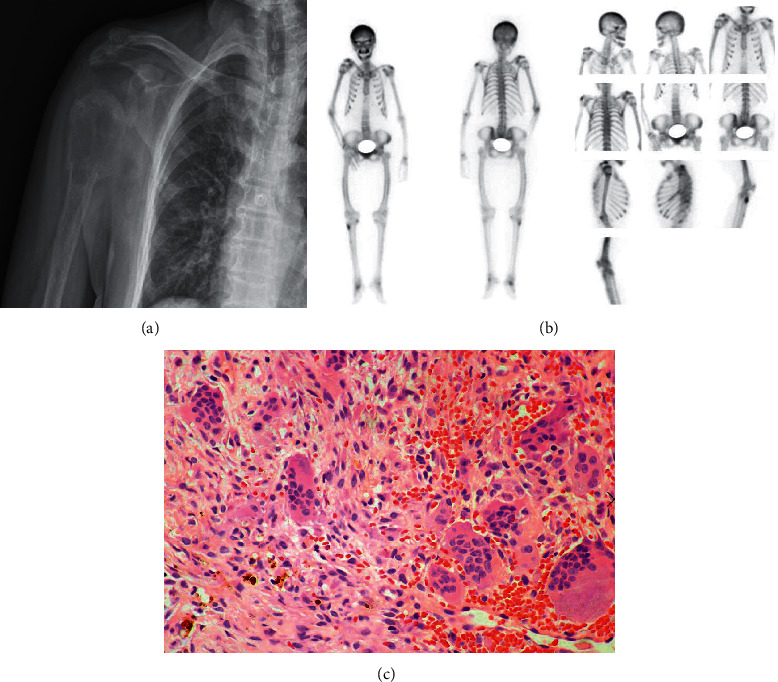
(a) Chestx-ray showing loss of cortical continuity through the surgical neck of the right proximal humerus with a lytic lesion, suggesting an impacted fracture and cortical alteration of the distalclavicle. (b) Bone scintigraphy with MDP-99 m Tc, shows the abnormal distribution of the radiotracer due to diffuse and generalized uptake increase in the bone system, with a reduction in soft tissues. (c) Proximal right humerus biopsy showing multiple giant osteoclast cells, dispersed among a fibrous stroma with hemorrhagic foci and hemosiderin deposits and scarce osteoid with prominent resorption by osteoclasts indicating compatible with Brown tumor due to hyperparathyroidism.

The orthopedics department ordered an X-ray to evaluate her. The X-ray (Figure 2(a)), showed a loss of cortical continuity through the surgical neck of the right proximal humerus and a lytic lesion, suggesting an impacted fracture and cortical alteration of the distal clavicle. On physical examination, shoulder girdle atrophy was observed, with intense pain on posterior palpation, and evident movement restriction of the shoulder. No other abnormalities were noted in the rest of the physical examination.

After consultation with the oncologic orthopedics department, the proximal humerus lesion was considered suggestive of metastatic cancer of unknown primary - thorax and abdomen. Computed tomography (CT) was performed, with evidence of expansive lytic lesion on the proximal humerus, sternum, acromion, sixth anterior left costal arch, iliac bones, left femoral head, and fracture of the superior vertebral disc (Supplementary Figure 1). Bone scintigraphy with MDP-99mTc showed a “superscan” (Figure 2(b)). A biopsy of the humerus lesion was obtained, demonstrating a brown tumor (Figure 2(c)). Additionally, biochemical tests showed parathyroid hormone-dependent hypercalcemia, hypophosphatemia, hypomagnesemia, and hyperchloremia (Table 1).

**Figure 3:**
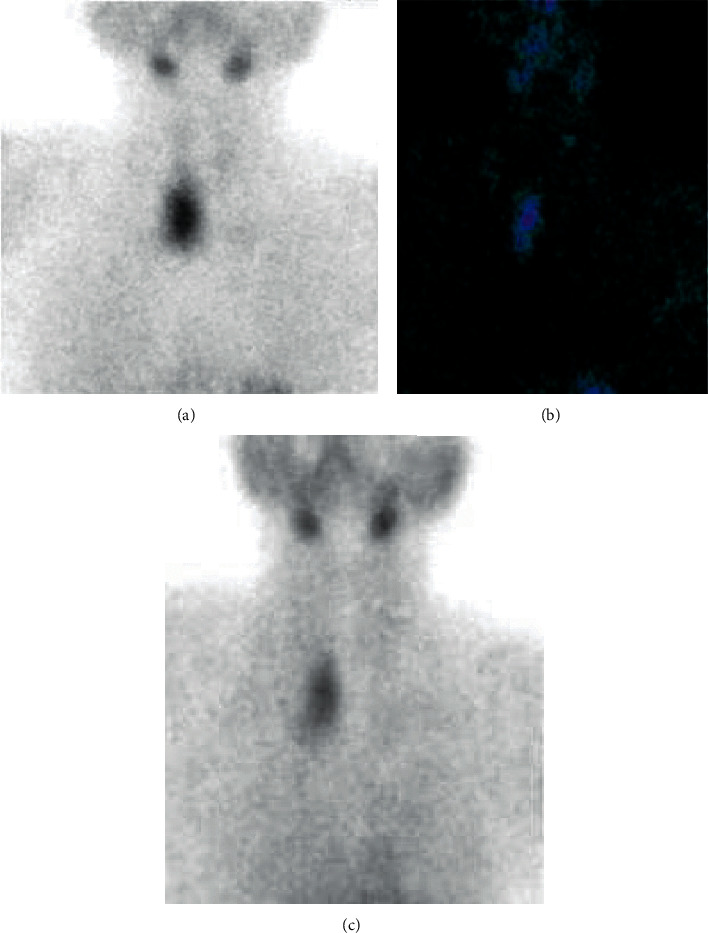
A, B, C. Scintigraphy showing a nodular goiter with uptake of right thyroid lobule, and suppressed uptake in the rest of the glandule.

**Figure 4:**
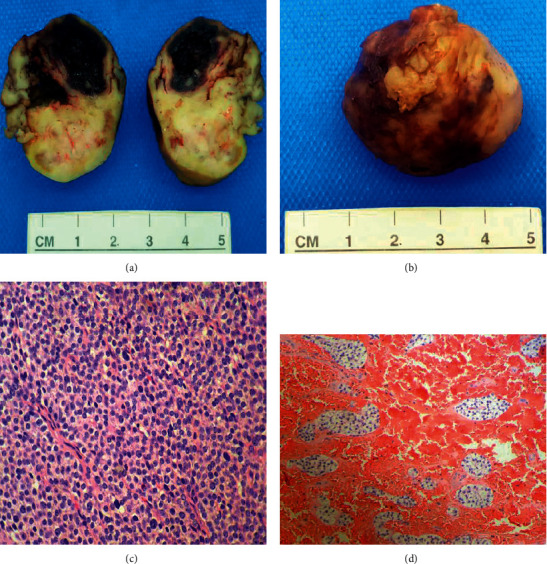
A, B, C, D. Biopsyo fright parathyroid mass. Mass showing 5 cm diameter and 16 grams weight. A neoplastic proliferation of parathyroid cells with areas of clear cells, oxyphilic cells, and chief cells. The lesion is profusely vascular with a hemorrhagicnet, with no frank necrosis. No macroscopical findings of malignancy.

With the diagnosis of PHPT along with skeletal involvement, 99 m Tc- sestamibi scintigraphy exhibited a nodular goiter with uptake in the right thyroid lobule (Figure 3). The patient underwent surgery, and a parathyroid tumor of 5 cm diameter and weight of 16 grams, compatible with parathyroid adenoma on histological evaluation was found (Figure 4). The patient recovered with no complications and was discharged on the fourth post-operatory day.

The patient was re-admitted 72 hours after discharge due to generalized myalgias, cramps in the lower limbs, perioral paresthesia and chest pressure. She also complained of dyspnea, tachycardia, tachypnea, with articular edema and pain mostly in the inner malleolus and proximal interphalangeal joints of both hands. Blood chemistry showed hypocalcemia and hypophosphatemia. A HBS diagnosis was made; calcium gluconate infusion was immediately started, with oral calcium and calcitriol administration, which led to symptomatic improvement. However, the patient persisted with inflammatory joint involvement consistent with pseudogout attack. Chondrocalcinosis was demonstrated and symptoms improved with prednisolone 10 mg/day and colchicine 0.5 mg/day administration. As the dyspnea and tachycardia prevailed, a D-dimer was performed - it came back on 5529 ng/mL (cut-off < 500 ng/ml). Anticoagulant therapy with dalteparin was initiated.

Simultaneously, a CT coronary angiogram was performed which confirmed acute and chronic pulmonary embolisms with lobar and segmentary branches involvement in both inferior pulmonary lobules. The clinical evolution was satisfactory. A month after discharge, the patient remains asymptomatic, with normal calcium, phosphorus, magnesium, and vitamin levels, serum PTH under 60 pg/mL, and urinary calcium of 25 mg (<4 mg/kg/24 h).

## 4. Discussion

Parathyroid adenomas are a well-known cause of PHPT. When the diameter exceeds 2 cm, a differential diagnosis between GTPA and parathyroid carcinoma (PC) must be performed [7]. One of the imaging parameters with a higher discriminatory capacity between these two entities is the depth/width relationship, with a value < 1 corresponding in 94% of cases to GTPA [8, 9]. Serum PTH level also correlates with the size of the adenoma: a serum level >232 ng/L corresponds to a 95% probability of *a* > 250 mg adenoma [9].

**Figure 5:**
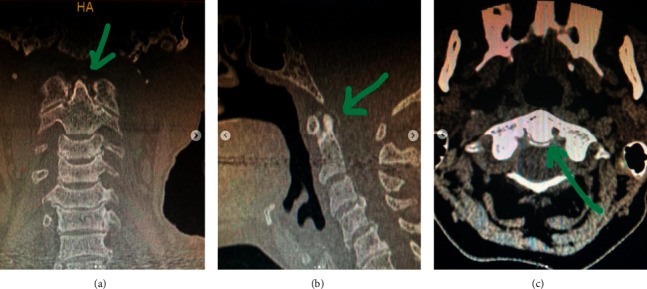
Cruciform ligament: “Crowned dens syndrome”.

Our patient presented a pathological fracture associated with osteitis fibrosa cystica (OFC). In a cohort of 74 patients with hyperparathyroidism, 40% presented with musculoskeletal complaints such as pseudogout, back pain, vertebral fracture, and arthralgias. Furthermore, in 20.2% of the patients, OFC was the most common radiological finding [10]. Until 2018, 30 cases of pseudogout after parathyroidectomy have been described [11]. In these cases, in most patients, the presence of chondrocalcinosis, and painful crisis were correlated with a decline in calcium and magnesium levels 24–48 hours after surgery. This finding matches the patient cohort described earlier that had chondrocalcinosis with acute or chronic arthritis and most developed a pseudogout crisis in the postoperative period [10]. Additionally, this patient presented with calcification of the cruciform ligament in a neck CT, also known as Crowned Dens syndrome (Figure 5), a finding that has been described in patients with chondrocalcinosis [12].

Similar to this case, neoplastic suspicion is the first differential diagnosis often reported in patients who are diagnosed with OFC and brown tumors [5]. This means hyperparathyroidism is an important differential diagnosis to consider early on in the diagnostic process to further decrease morbidity. Parathyroidectomy improves the quality of life, bone mineral density, and reduces risks of fracture and nephrolithiasis [1].

This patient developed one of the most fearsome complications of parathyroidectomy - HBS, observed in up to a fourth of patients who undergo parathyroidectomy due to secondary hyperparathyroidism in the USA. They are re-admitted in the first 30 days because of hypocalcemia (incidence of 28%), however, less precise data has been reported in primary hyperparathyroidism (between 4 and 87%) [13].

Al-Hassan et al. have previously described a relationship between the size of GTPA and the incidence of HBS in a case series [4]. In our case, the adenoma had a 16 g weight, considerably higher than the weight described as a risk factor for HBS development (Table 2 and Supplementary Table 1). Other findings related to HBS include markedly elevated PTH and alkaline phosphatase (AF) levels (although the evidence is scarce and no specific cut off point has been established). Most case series also report *a* >1000 pg/mL for PTH and >3 times the upper normal limit for AF [14]. Other factors include a high count of osteoclasts in bone biopsy and the presence of brown tumors as well as OFC, all of which were present in our patient. Furthermore, a serum calcium level drop below 8.4 mg/dL has been observed on the second to fourth postoperative days in patients who develop HBS, associated with hypomagnesemia and hypophosphatemia, just as observed in our patient [13].

HBS treatment must be initiated as soon as the diagnosis is done, as these patients can develop severe complications that increase morbidity and mortality [15]. It is recommended to administer a dose of 4–12 g/day of elemental calcium and a dose of 2–4 ug/day of calcitriol through an oral route to achieve an improvement of calcium levels [13], as was performed in the described case. Furthermore, the use of IV calcium salts has been described to achieve rapid resolution of severe symptoms of neuromuscular irritation along with replenishing magnesium and phosphorus when needed [15]. Nonetheless, preventive measures are vital to avoid this complication, such as the prophylactic administration of calcium and vitamin D the day before surgery, and bisphosphonate use, both reduce in-hospital stance and the drop in calcium levels [15]. In a systematic review performed by Sanabria et al. [16], a useful strategy was found to reduce symptomatic hypocalcemia - the prophylactic administration of calcium plus vitamin D in the postoperative period, even in those patients with normal calcium levels post parathyroidectomy. This strategy could reduce in up to 70% the transient symptomatic hypocalcemia, compared to patients who do not receive such prophylaxis.

In our patient, normocytic-normochromic anemia was notable, which improved after disease treatment and hyperparathyroidism resolution; anemia in a patient with primary hyperparathyroidism is multifactorial, however, three mechanisms have been described to explain the pathophysiology. First, a direct inhibition of erythroid line growth by PTH which, in cell cultures, improves when erythropoietin (EPO) is administered [17]. Second is the peripheral destruction of erythrocytes mediated by the alteration of calcium metabolism, an increase in membrane fragility, and the increase of cytosolic calcium [18]. The third and final one is medullary fibrosis secondary to hyper para thyroidism, given the potent effect of PTH on fibroblasts. Prospective studies show that patients with primary hyperparathyroidism and normocytic-normochromic anemia may have medullary fibrosis in up to 75% of cases–demonstrated through bone marrow aspirate, and such fibrosis and anemia improve when parathyroidectomy is performed [17, 18]. It is worth remarking that medullary fibrosis is not associated with disease timespan, hypercalcemia severity, or PTH levels [19], contrary to anemia in secondary hyperparathyroidism due to chronic kidney disease. Our patient's red blood cell count improved after surgery.

Another complication our patient developed was pulmonary embolism. The association between hypercalcemia and thrombosis has been described in literature. However, the evidence is scarce and the direct association with PHPT is still controversial [20]. In a letter to the editor, Franchello et al. described a retrospective cohort of 60 patients with acute hypercalcemia (defined as calcium levels >15 mg/dL), of which, 11.7% presented with a major thrombotic event (cerebrovascular disease, pulmonary embolism, deep vein thrombosis) [21]. Based on this, a hypothesis was formulated which stated that, the dehydration state secondary to hypercalcemia, the nephrogenic diabetes insipidus, the proinflammatory state of hypercalcemia, and the vasoconstriction mediated by calcium may be responsible for the hypercoagulability state [21].

In conclusion, PHPT due to GTPA is a disease that can develop bony lesions that may be confused as metastatic lesions of unknown primary. Also, the incidence of postoperative complications such as HBS seems to be greater compared to PA. A correct clinical and laboratory evaluation may clear the diagnosis of a curable disease and could prevent morbidity and mortality in these patients.

## Figures and Tables

**Table 1:** Laboratory results during patient's hospital stay.

Laboratory test	Results	Reference value
Hemoglobin	9.2	12–15.5 gr/dL
Leucocytes/Neutrophils/lymphocytes	9400/6200/2200	4.000–11.000 × mm^3^/2000–7000/1000–3000
Platelets	315.000	200.000–500.000/mm3
ESR	7	1–20 mm/h
Aspartatotransaminasa (AST)/ Alaninatransaminasa (ALT)	17/15	10–40 units/L/7–56 units/L
Total bilirrubin/direct bilirrubin	0.3/0.03	0.1–1.2 mg/dL/<0.3 mg/dL
Alkaline phosphatase	814	20–140 IU/l/9–48 units/L
Albumin	3.8	3.4–5.4 g/dL
Lactate dehydrogenase	136	140–280 U/L
Ferritin	337	20–200 ng/mL
Na +/K+/Cl − /Mg	141/3.3//114/1.2	135–145/3.6–5.2/96–106/1.7–2.2 mEq/L
TP/TPT	12.4/21	10–12 s/25–35 s
Creatinin	1.08	0.7–0.3 mg/dL
Blood urea nitrogen	26	8–20 mg/dL
Vitamin D	14.9	25–65 pg/mL
24 h Urine Ca2+	550	100–300 mg/24 h
Parathyroid hormone	1973	10–65 pg/mL
Calcium	14.9	8.5–10.5 mg/dl
Phosphorus	3	2.8–4.5 mg/dL

**Table 2:** Patients with giant parathyroid adenoma, clinical characteristics, tumor pathologies and postoperative complications.

Case report	Gender	Age (Years)	Side	Presentation	Ca (mg/dL)/PTH (ng/L)	Dimensions (mm)	Weight (g)	Pathology	Post-operative complications
Aggarwal et al., 2009 [22]	F	33	L	Edema, palpable nodule, bone pain, R humerus and R pelvic fracture	10.6/762	95 × 50 × 35	102	Chief cell adenoma	Symptomatic hypocalcemia

Salehian et al., 2009 [23]	F	53	R	Edema, bone pain, weight loss, nausea, vomit	14.63/1624	55 × 35 × 20	30	PTA	No complication

Sisodiya et al., 2011 [24]	F	52	R	Recurrent vomit	17.03/598	39 × 20 × 17	ND	ND	Hypocalcemia

Asghar et al., 2012 [25]	F	55	L	Parathyroid crisis, palpable nodule	23.05/1182	110 × 70 × 60	ND	Cystic degeneration PTA, no lymph node metastasis	No complication

Vilallonga et al., 2012 [26]	F	19	L	Parathyroid crisis	14.23/1207	Maximum diameter 30	70	Intrathyroid PTA	No complication

Neagoe et al., 2014 [27]	M/F/F	57/60/33	R/L/R	C 1: bone pain, abdominal pain, nausea, palpable nodule. C 2: parathyroid crisis, palpable nodule. C 3: recurrent renal calculi, brown tumor in tibia	C 1 : 3.54/1780 C 2 : 16.19/863 C 3 : 12.63/1174	C 1 : 50 × 30 × 20 C 2 : 55 × 40 × 30 C3ND	C 1 : 30.6 C 2 : 35.2 C 3:>30	2 PTA; 1 partially cystic PTA	C 1: hungry bone syndrome C 2: mild hypocalcemia and hungry bone syndrome C 3: mild hypocalcemia

Haldar et al., 2014 [28]	F	61	L	Asymptomatic	12.71/179.2	65 × 30 × 15	12	PTA	No complication

Garas et al., 2015 [29]	F	53	L	Palpable nodule, bone pain	15.95/4038	Maximum diameter 70	27	Chief cell PTA	No complication

Case report	Gender	Age (Years)	Side	Presentation	Ca (mg/dL)/PTH (ng/L)	Dimensions (mm)	Weight (g)	Pathology	Post-operative complications

Rutledge et al., 2016 [30]	F	21	R	Neck mass enlargement, constipation, palpable nodule	10.94/1305.1	80 × 55 × 30	58.8	Atypical PTA	Symptomatic hypocalcemia, hungry bone syndrome

Krishnamurthy et al., 2016 [31]	M	50	L	Recurrent acute pancreatitis crisis, palpable neck mass	11.1/669	Maximum diameter 60	20	PTA	Hipocalcemia

Castro et al., 2017 [32]	F	40	L	Asymptomatic palpable nodule	13.43/825	64 × 16 × 20	10.8	PTA	Hypocalcemia

Sahsamanis et al., 2017 [33]	F	42	L	Abdominal pain	10.42/151	33 × 20 × 14	5.39	PTA	No complication

Mantzoros et al., 2018 [34]	F	73	R	Enlargement of neck size, bone pain	14.55/1629	50 × 25 × 25	30	PTA	Hungry bone syndrome

Migliore et al., 2013 [35]	F	65	R	Persistent hypercalcemia	Both elevated	―	95	PTA	No complication

Taghavi Kojidi et al., 2016 [36]	M	70	Middle	Anorexia, nausea, bone pain, constipation, symptomatic nephrolithiasis, polydipsia	14.55/930	―	75	Active parathyroid lesion	Hypocalcemia

Pecheva et al., 2016 [37]	F	72	R	Depression, severeosteoporosis (*T* = − 3,2)	12.1/250.8	―	19	PTA	Hoarseness, cough

Talukder et al., 2017 [38]	F	49	Middle	Brown tumor	14.07/1000	40 × 30 × 20	12	Neuroendocrine cell tumor	No complication

Garuna Murthee et al., 2018 [39]	M	72	Middle	Anorexia, lethargy, abdominal cramps, constipation, weight loss	15.19/1867.1	Maximum diameter 78	220	Intrathymic PTA	No complication

Miller et al., 2019 [40]	M	53	Middle	Asymptomatic nephrolithiasis	11.22/179.2	80 × 30 × 30	30.9	PTA	No complication

P liu et al., 2020 [41]	M	79	L/R	Obstructive right nephropathy, and acute kidney injury secondary to hypercalcemia	13.19/420.5	L: 50 × 25 × 12 R: 48 × 22 x 10	L:8101 R:7339	PTA	No complication

Giuseppe Evola et al., 2020 [42]	F	52		Musculoskeletal pain, constipation, polyuria, pathological fracture of bilateral femur	12.5/27.47	65 × 50 x 30	90	PTA	No complication

Alvarez JC et al., 2021 [our case]	F	55	R	Polyarthralgias, pathological fracture of humerus, brown tumor of humerus	14.9/1973	Maximum diameter 5	16	PTA	Hungry bone syndrome

## Supplementary Materials

Supplementary Materials Supplementary Figure 1A, BLytic lesion which involves lunate, hamate, and distal pole of the scaphoid bone bilaterally. Lytic lesion in distal radius. Generalized demineralization with thickening of trabecular pattern. Moth-eaten pattern (subperiosteal resorption) in metacarpal and phalanxes. Furthermore, cortical is irregular with small lytic images in the distal portion of the visible phalanxes. C, D Skull with salt and pepper lesions, a fine speckled pattern, and loss of the internal/external cortical definition. Supplementary Table 1 Characteristics of case reports with giant parathyroid adenomas.
