# Supplementary material for: Giant Parathyroid Adenoma-Associated Fracture, Not All Lytic Bone Lesions are Cancer: A Case-Based Review
Source: Case Rep Med. 2022 Jan 29;2022:3969542. doi: 10.1155/2022/3969542 (PMC8817849; doi:10.1155/2022/3969542)
Supplement: Supplementary Materials — Supplementary Figure 1A, BLytic lesion which involves lunate, hamate, and distal pole of the scaphoid bone bilaterally. Lytic lesion in distal radius. Generalized demineralization with thickening of trabecular pattern. Moth-eaten pattern (subperiosteal resorption) in metacarpal and phalanxes. Furthermore, cortical is irregular with small lytic images in the distal portion of the visible phalanxes. C, D Skull with salt and pepper lesions, a fine speckled pattern, and loss of the internal/external cortical definition. Supplementary Table 1 Characteristics of case reports with giant parathyroid adenomas. [file 3969542.f1.docx]

**Supplementary Material**

**A**


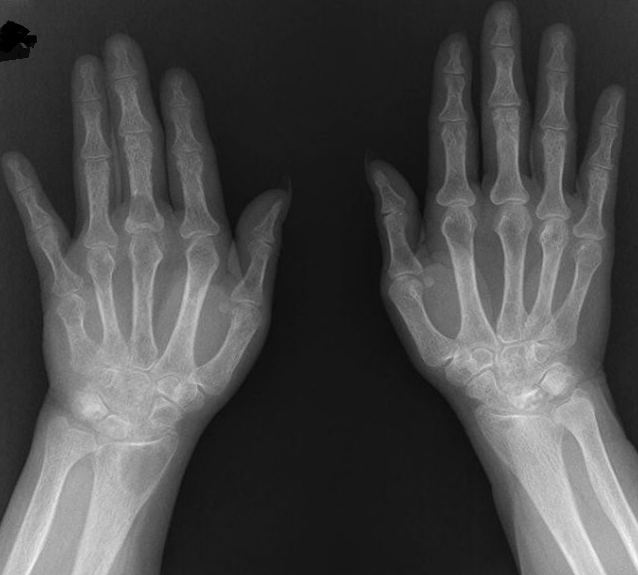


**B**


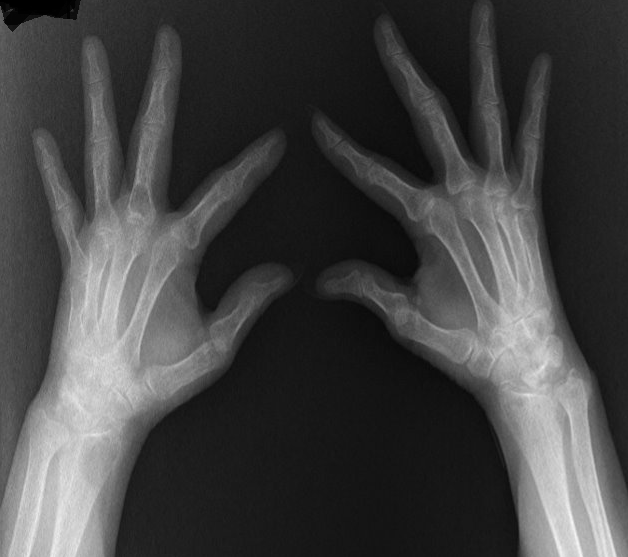


**C**

**
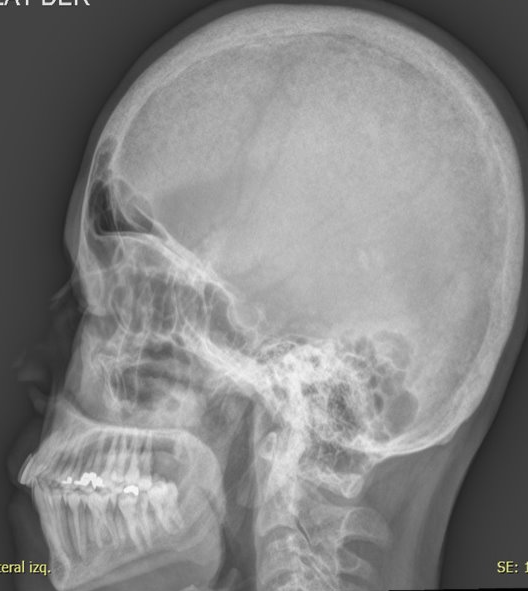
**

**D**

**
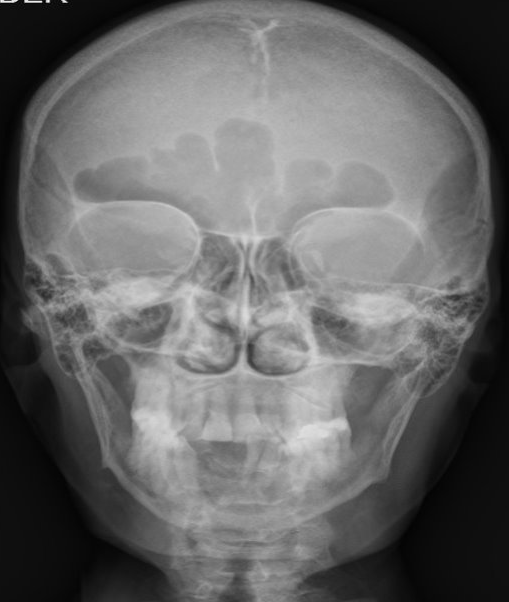
**

**Supplementary figure 1. A, B** Lytic lesion which involves lunate, hamate, and distal pole of the scaphoid bone bilaterally. Lytic lesion in distal radius. Generalized demineralization with thickening of trabecular pattern. Moth-eaten pattern (subperiosteal resorption) in metacarpal and phalanxes. Furthermore, cortical is irregular with small lytic images in the distal portion of the visible phalanxes. **C, D** Skull with salt and pepper lesions, a fine speckled pattern, and loss of the internal/external cortical definition.

| **Case reports from 2009-2020 (n =24)** | **Findings** |
| --- | --- |
| Gender | ♀70% |
| Age | Mean 52 years (24-78) |
| Side | Left 45% Right 35% Middle 20% |
| Clinical presentation | Palpable nodule 45%  Bone pain 33%  Brown tumor 12.5%  Nephrolitiasis 12.5%  Constipation 25%  Asymptomatic 12.5%. |
| Metabolic profile | Mean Ca 13.8 mg/dl Mean PTH 1109 ng/L |
| Tumor weight (g) | Mean 47.24 g |

| Post-operative complications | Symptomatic hypocalcemia (32%)  Hungry bone syndrome (25%): especially with weight ≥15g |
| --- | --- |

**Supplementary Table 1.** Characteristics of case reports with giant parathyroid adenomas.
